# Transcriptional response of grapevine to infection with the fungal pathogen *Lasiodiplodia theobromae*

**DOI:** 10.1038/s41598-019-41796-9

**Published:** 2019-03-29

**Authors:** Wei Zhang, Jiye Yan, Xinghong Li, Qikai Xing, K. W. Thilini Chethana, Wensheng Zhao

**Affiliations:** 1 State Key Laboratory of Agrobiotechnology and MOA Key Lab of Pest Monitoring and Green Management, China Agricultural University, Beijing, 100193 China; 2 Institute of Plant and Environment Protection, Beijing Academy of Agriculture and Forestry Sciences, Beijing, 100097 China; 3 Beijing Key Laboratory of Environment Friendly Management on Fruit Diseases and Pests in North China, Beijing Academy of Agriculture and Forestry Sciences, Beijing, 100097 China

## Abstract

Botryosphaeria dieback on the grapevine is caused by Botryosphaeriaceae fungi, which threatens the yield and quality of grapes. At present, chemical control strategies are often observed to be ineffective in controlling the disease worldwide. Improving our understanding of the molecular mechanisms that confer resistance to pathogens would facilitate the development of more pathogen-tolerant grape varieties. Here, we used RNA sequencing analysis to profile the transcriptome of grapevine green shoots infected with *Lasiodiplodia theobromae* over a time course of 4, 8 and 12 hours post inoculation. A total of 5181 genes were identified as differentially expressed genes (DEGs), and DEGs were more abundant over time. Further analysis revealed that many of these DEGs are involved in plant-pathogen interactions, hormone signal transduction and phenylpropanoid biosynthesis pathways, suggesting that innate immunity, phytohormone signaling and many phenylpropanoid compounds, which constitute a complex defense network in plants, are involved in the response of grapevine against to *L*. *theobromae* infection. This study provides novel insights into the molecular mechanisms of plant–pathogen interactions that will be valuable for the genetic improvement of grapevines.

## Introduction

Grapes is one of the most cultivated fruits around the world, and fungal diseases are very serious threats to its production and quality. Grapevine Botryosphaeria dieback caused by species in the family Botryosphaeriaceae has become an economically important problem in almost all main grapevine growing countries in the last few decades^1–4^, with annual losses of more than $260 million USD in the state of California (USA) alone^5^. More than 20 Botryosphaeriaceae species are associated with grapevine Botryosphaeria dieback, and the most common symptoms are cankers and fruit rot^1,6,7^. However, the virulence and symptoms caused by a particular Botryosphaeriaceae species can differ greatly between grapevines based on geographical location^3,8–12^. Botryosphaeriaceae species are reported to infect their host through wounds or natural openings, such as lenticels and stomata^13–15^. These fungi can remain in a latent phase as endophytes without inducing apparent symptoms after entering their hosts and then change to the pathogenic phase as plant opportunistic fungal pathogens to cause disease symptoms when the hosts are stressed^16–18^. It is thus very difficult to determine the infection status of a host and conduct timely control strategies. Recent genomic and transcriptomic analyses have shown that these pathogens have evolved special adaptive mechanisms to infect woody plants^15,18^. These mechanisms include significant expansions of gene families associated with virulence and nutrient uptake, including cellular transporters, cell wall degrading enzymes (CWDEs), cytochrome P450s, putative effectors and biosynthesis of secondary metabolites. Furthermore, potential virulence functions of CAzymes enriched in *Lasiodiplodia theobromae*, which play crucial roles in early colonization and pathogenesis, were also revealed. Thus, understanding the molecular responses of grapevine to fungal infection could enable the development of disease-resistant cultivars as an effective and environmentally friendly strategy to control diseases.

To date, our knowledge of host defense responses to pathogens mainly comes from studies in the plant-pathogen model systems, such as Arabidopsis, tomato, tobacco and rice^19–22^. These studies have mentioned that the plant defense response is a complex network, including innate immunity, multiple signaling pathways, secondary metabolic products, and cross talks. Nonetheless, information on the host responses to the plant opportunistic fungal pathogens in woody tissues of perennial plants is very limited^23^. Most studies on woody tissues responding to pathogen infection have focused on the plant’s physical responses or metabolic changes, such as formation of tyloses and gels in xylem^24^, cell wall modifications with suberin^25^, production of resins^26^, and accumulation of peroxidases, superoxide dismutases, glutathione S-transferases, phenolic compounds, stilbenes, and phytoalexins^20,27–32^. Recently, defense responses, for example, cell death, expression of pathogenesis-related (PR) genes, and alkalinization of extracellular medium, were observed to be induced by purified secreted proteins isolated from two Botryosphaeriaceae species, *Neofusicoccum parvum* and *Diplodia seriata*, in *Vitis* suspension cells^33^. In recent decades, RNA sequencing (RNA-seq) has enabled the study of the transcriptional responses of woody plants to phytopathogens, revealing that genes involved in cell wall biosynthesis, hormone signal transduction and secondary metabolism processes play important roles^34–37^.

In the present study, we performed a transcriptome analysis of grapevine (*Vitis vinifera*) infected by *L*. *theobromae*, the most aggressive Botryosphaeriaceae species on grapevines in China^3^. Our objectives were to gain overall insight into the grapevine-*L*. *theobromae* interaction and identify the genes associated with key defense-related responses in grapevine during the early infection stages of *L*. *theobromae*. The comprehensive analysis of gene expression in this study will provide a better understanding of the resistance mechanism of grapevine against to Botryosphaeriaceae species and facilitate future research on the application of genomics-assisted breeding systems in grapevine.

## Results

### Sequencing data summary and prediction of novel transcripts

*Lasiodiplodia theobromae* successfully infected grapevine green shoots and produced visible lesions within 24 hours after inoculation. However, the defense mechanism of grapevine against fungal diseases is not very clear until now. Therefore, to understand the response of grapevine against *L. theobromae* during the early infection stages, we used Illumina next-generation sequencing technology (Illumina, San Diego, CA, USA) to analyze the transcriptomes of grapevine green shoots that had been inoculated with *L. theobromae* after 0 (control), 4, 8, and 12 hours post-inoculation (hpi), with two biological replicates in each of the four treatments (Supplementary Fig. S1; Supplementary Table S1). In total, we generated 135.68 MB reads with an average of 16.96 MB for each library. After strict filtration, ranging from 12.46 to 22.11 MB, the high-quality reads were mapped to the grape genome for further gene expression analysis (Supplementary Table S2). The high-quality reads were deposited in the NCBI SRA database under accession number SRP134225. We optimized the structure of 7,200 genes using the 29,971 annotated genes in the grape reference genome. In addition, we predicted 211 novel transcripts and quantified the expression levels of 13,354 genes (including 177 novel transcripts) across all eight plant samples (Supplementary Table S3).

### Global gene expression profiling analysis and identification of differentially expressed genes (DEGs)

The correlation coefficient between two replicate samples was determined based on the FPKM values of genes. The R^2^ values of the four replicate groups (each representing one time point after inoculation) were 0.955, 0.962, 0.960 and 0.973. These results suggest that the replicate samples exhibited high repeatability and reliability. This result ensures our ability to obtain reliable differences in gene expression analyses between different *L. theobromae*-inoculated treatments and the 0 hpi sample (Supplementary Fig. S2).

**Figure 1:**
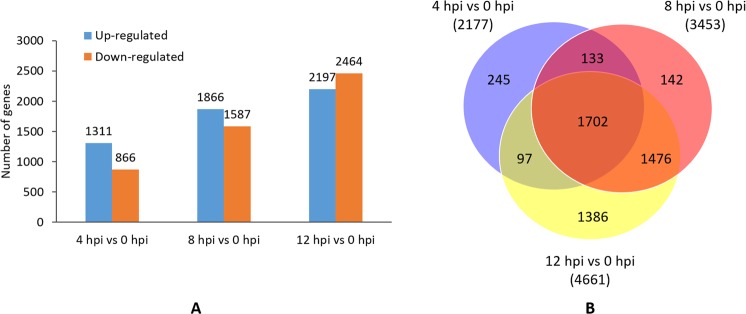
Identification of differentially expressed genes (DEGs) of grapevine infected with *Lasiodiplodia theobromae* by pairwise comparison of eight transcriptomes: (**A**) The up- and down-regulated DEGs at 4, 8, and 12 hours post-inoculation (hpi). (**B**) Venn diagram displaying the distribution of the DEGs at different time points.

To explore the differences among the transcriptomes induced at different time points after inoculation, we performed separate pairwise comparisons between each of the three *L*. *theobromae*-inoculated treatments and the 0 hpi sample. In total, 5181 genes were identified as DEGs with a |log2 fold change| > 2.0 and an adjusted p-value < 0.05 (Fig. 1A, Supplementary Tables S4–S7). Among these genes, 2,177 (1,311 up-regulated and 866 down-regulated), 3453 (1866 up-regulated and 1587 down-regulated) and 4661 (2197 up-regulated and 2464 down-regulated) DEGs were identified in 4 hpi vs 0 hpi, 8 hpi vs 0 hpi and 12 hpi vs 0 hpi, respectively (Fig. 1A). Groups of 245, 142, and 1,386 DEGs were identified as unique to 4 hpi vs 0 hpi, 8 hpi vs 0 hpi and 12 hpi vs 0 hpi, respectively, while 1,702 common genes were regulated at all three time points. A total of 1,706 genes were commonly expressed between at least two time points, with 133 genes between 4 hpi and 8 hpi, 1,476 between 8 hpi and 12 hpi, and 97 between 4 hpi and 12 hpi (Fig. 1B). The number of DEGs increased with time after inoculation. The 12 hpi treatment induced both the greatest number of DEGs and the maximum unique DEGs among the three time points. The unique DEGs expressed at different infection time points may play specific roles at the related infection time point.

**Figure 2:**
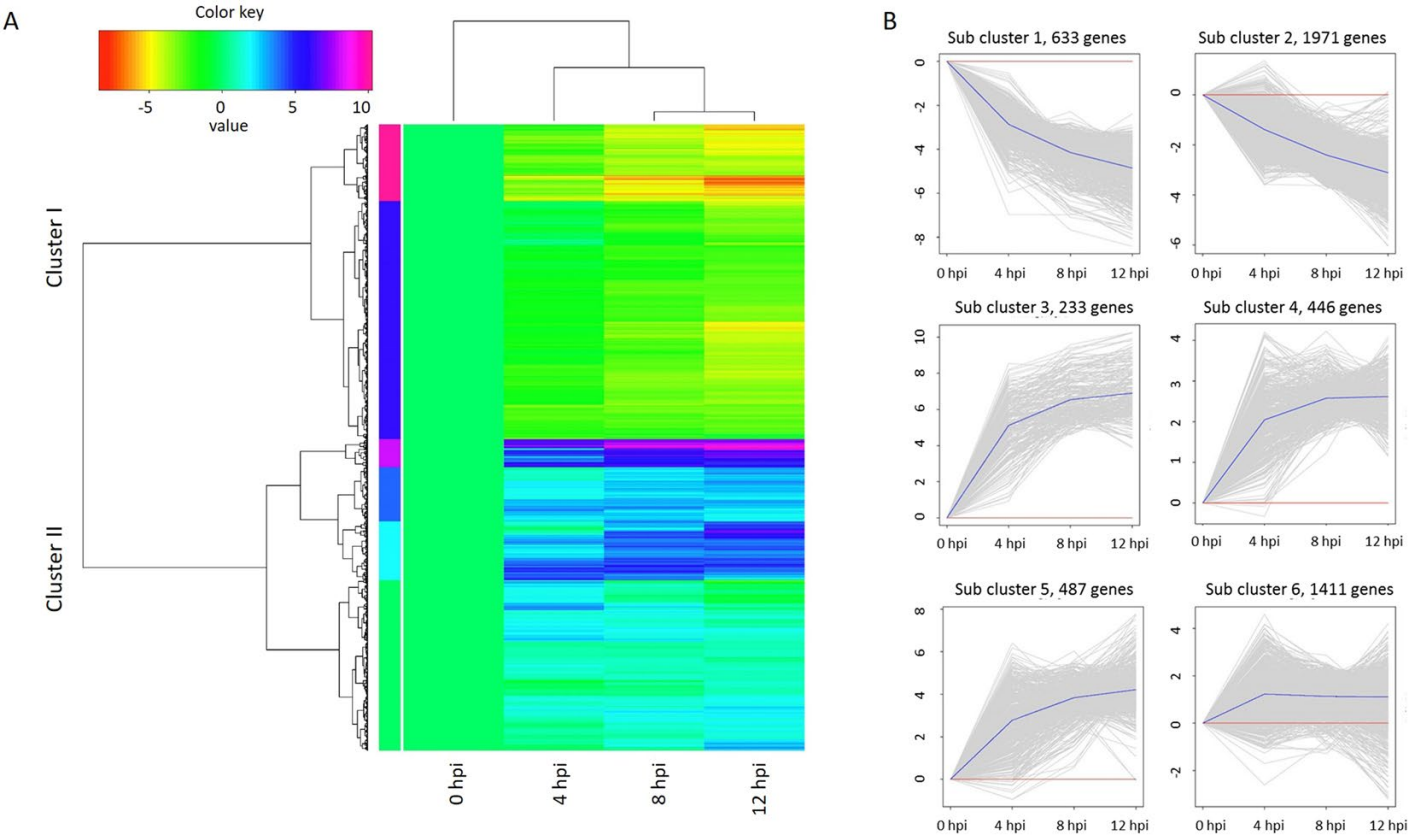
The expression patterns of all DEGs of grapevine over time: (**A**) Heat map showing the expression changes of all DEGs at each time point. Cluster I and cluster II represent down-regulated and up-regulated DEGs, respectively. (**B**) Expression trends of genes in six sub-clusters according to the time course. The y-axes represent the gene expression level, and the x-axes represent different time points after *L*. *theobromae* infection. Thin gray lines represent the expression levels of individual genes. Blue lines represent the average expression level of genes in the subcluster. The number of genes in each sub-cluster is indicated.

To better understand the transcriptome changes in response to *L*. *theobromae* infection in grapevine, the expression patterns of all DEGs were analyzed. The expression patterns of all DEGs across the three inoculated time points indicated that the DEGs could be grouped into two main clusters (cluster I and cluster II) and further into six sub-clusters (Fig. 2). Approximately half (50.26%) of the DEGs belonged to cluster I and were down-regulated, whereas the remaining 49.74% of the DEGs, denoted cluster II, were up-regulated after infection (Fig. 2A). Notably, abundant DEGs were sharply induced at 4 hpi.

### Gene Ontology classification and Kyoto Encyclopedia of Genes and Genomes analysis of DEGs

The functional categories of all DEGs were analyzed based on Gene Ontology (GO) annotation to gain functional information. In total, 1744 of 2177 DEGs in 4 hpi vs 0 hpi, 2832 of 3453 DEGs in 8 hpi vs 0 hpi, and 3766 of 4661 DEGs in 12 hpi vs 0 hpi were assigned to at least one term of the three major categories: biological process (BP), molecular function (MF) and cellular component (CC) (Supplementary Table S8). Of the 1,702 common DEGs, 1,382 were categorized into at least one GO term, among which 788 were up-regulated, whereas 594 were down-regulated. A large number of the up-regulated common DEGs were associated with catalytic and binding activity in the MF category and were found to participate in metabolic processes, defense responses, and secondary metabolic processes in the BP category.

A Kyoto Encyclopedia of Genes and Genomes (KEGG) enrichment analysis was performed to identify the biochemical pathways in which the DEGs were involved in grapevine (organism code “vvi”). The common DEGs were involved in both primary and secondary metabolisms, such as biosynthesis of amino acids (vvi 01230), biosynthesis of secondary metabolites (vvi 01110), carbon fixation in photosynthetic organisms (vvi 00710), carbon metabolism (vvi 01200), circadian rhythm-plant (vvi 04712), flavonoid biosynthesis (vvi 00941), glycolysis/gluconeogenesis (vvi 00010) and ribosome (vvi 03010), which were significantly enriched (adjusted p-value < 0.05). Moreover, several pathways related to disease defense response were also enriched early in infection. Three of these pathways are detailed below.

### DEGs involved in the plant-pathogen interaction pathway

**Figure 3:**
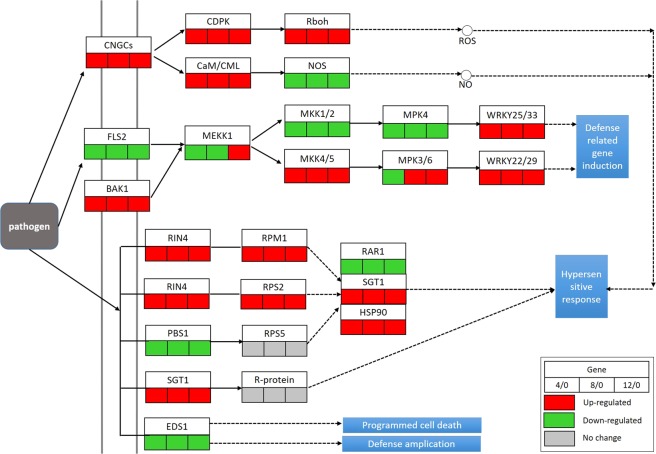
DEGs involved in the plant–pathogen interaction pathway in grapevine infected with *L*. *theobromae* based on KEGG analysis.

According to the KEGG pathway analysis, 83 DEGs identified in grapevine inoculated with *L. theobromae* were involved in the plant-pathogen interaction pathway, among which 32 were commonly differentially expressed across the time points (Fig. 3; Table S9). Cytoplasmic Ca^2+^ concentration rapidly accumulates when plants perceive pathogen-associated molecular patterns (PAMPs). The calcium signal is then transmitted by the calcium-dependent protein kinase (CDPK) and calmodulin (CaM)/calmodulin-like proteins (CML) to produce reactive oxygen species (ROS) and nitric oxide (NO) separately through a sequence of biochemical reactions to induce defense responses^38^. Here, we observed that cyclic nucleotide gated channels (CNGCs), CDPK and CaM/CML were up-regulated after *L. theobromae* infection, whereas the expression of genes encoding nitric oxide synthase was down-regulated. Typically, once plants recognize PAMPs, a tandem of mitogen-activated protein kinase (MAPK) cascades are activated followed by WRKY transcriptional factors, which prompt expression of defense-related genes^21,39^. We identified nine DEGs: flagellin-sensing 2 (FLS2), brassinosteroid insensitive 1-associated kinase 1 (BAK1), mitogen-activated protein kinase kinase 1 (MEKK1), mitogen-activated protein kinase 1 (MKK1/2), mitogen-activated protein kinase 4/5 (MKK4/5), mitogen-activated protein kinase 4 (MPK4), mitogen-activated protein kinase 3/6 (MPK3/6), WRKY transcription factor 22 (WRKY22/29), and WRKY transcription factor 33 (WRKY 25/33), respectively. All of these orthologs were reported to be involved in the pathogen-associated molecular pattern (PAMP)-triggered immunity (PTI) pathway. Among these DEGs, three MAPKs and two WRKYs were up-regulated upon *L. theobromae* infection (Fig. 3).

Plants can employ a second layer of defense response to avoid the invasion of pathogens, which is known as the effector-triggered immunity (ETI) pathway^21,39^. We identified eight DEGs in the ETI pathway upon *L*. *theobromae* infection. Among the ETI genes that were up-regulated were one negative regulator PRM1-interacting protein 4 (RIN4), two R genes (RPM1 and RPS2), and two disease-resistance associated genes (SGT1 and HSP90). Conversely, a disease resistance protein (PBS1) and an enhanced disease susceptibility protein (EDS1) were down-regulated.

### DEGs involved in the plant hormone signal transduction pathway

**Figure 4:**
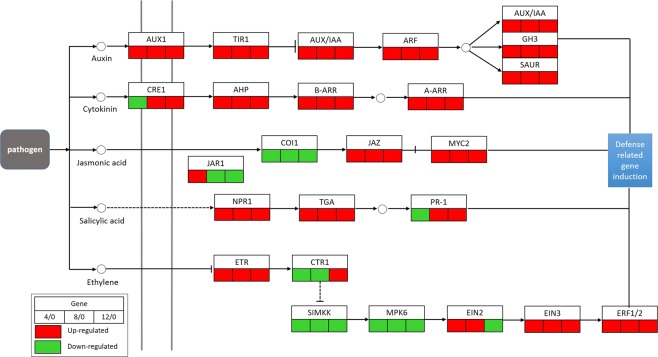
DEGs involved in the plant hormone signal transduction pathway in response to *L*. *theobromae* infection enriched by KEGG analysis.

Phytohormones act as critical signals for activating disease resistance in plants^40^. In this study, the signal transduction pathways of some main plant hormones such as auxin, cytokinin, jasmonic acid (JA), salicylic acid (SA), and ethylene (ET) were affected by *L. theobromae* infection. A total of 127 DEGs involved in the plant hormone signal transduction pathway were detected (Fig. 4, Supplementary Table S10). At 4 hpi, three DEGs in the JA transduction pathway were up-regulated (jasmonate ZIM-domain containing protein (JAZ), jasmonic acid-amino synthetase 1 (JAR1) and transcription factor MYC2). However, JAR1 was down-regulated at 8 hpi and 12 hpi, while JAZ and MYC2 continuously increased over time.

SA is an important plant hormone that regulates plant defense response^40^, and NPR1 is a key regulator of SA-mediated signal transduction. We found that NPR1 was up-regulated after *L*. *theobromae* infection. TGA transcription factors, which activate pathogenesis-related (PR) genes, such as PR-1, showed similar expression patterns with NPR1, whereas PR-1 was down-regulated at 4 hpi and then up-regulated at 8 hpi and 12 hpi. ET is another crucial phytohormone for inducing plant defense response^41^. We identified seven DEGs in the ET transduction pathway. Ethylene receptor (ETR), ethylene-insensitive protein 3 (EIN3), and ethylene-responsive factors were all up-regulated by *L*. *theobromae*. Nevertheless, MPK6 was down-regulated at all three time points. The genes encoding serine/threonine-protein kinase CTR1 were down-regulated at 4 hpi and 8 hpi but up-regulated at 12 hpi. Ethylene-insensitive protein 2 (EIN2) showed the opposite expression pattern of CTR1.

Auxin and cytokinin are associated with plant defense response^42^. In our study, we identified and analyzed 11 DEGs in the auxin and cytokinin signaling pathways. Ten out of 11 DEGs were up-regulated across all three treatment time points, and one, cytokinin response 1 (CRE1), was down-regulated at 4 hpi and subsequently up-regulated at 8 hpi and 12 hpi (Fig. 4).

### DEGs involved in the phenylpropanoid biosynthesis pathway

**Figure 5:**
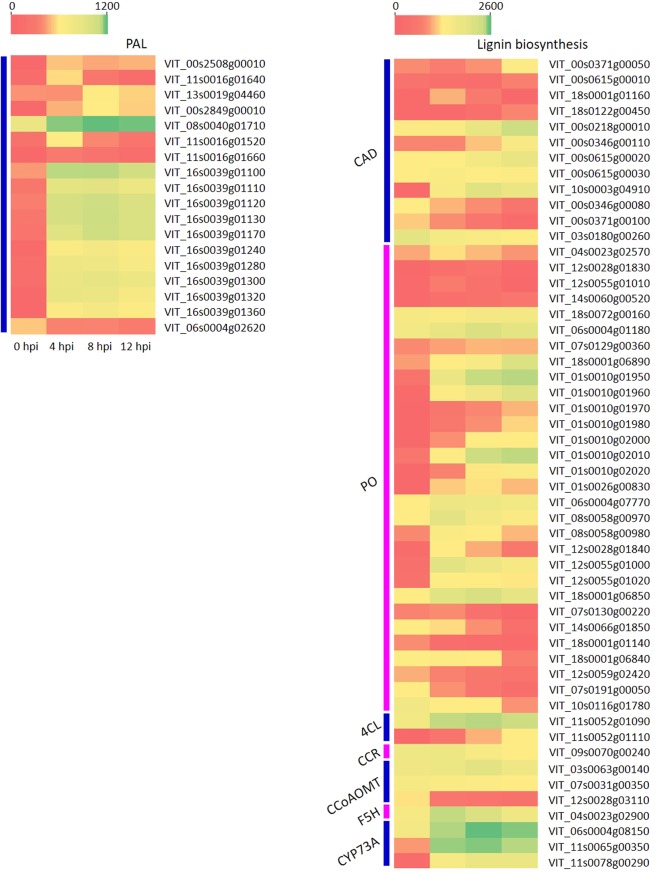
Expression patterns of representative DEGs in the phenylpropanoid biosynthesis (PB) pathway. The fragment per kilobase per million (FPKM) value was used to estimate the gene expression level. Heat map visualizing the expression patterns of the representative DEGs involved in the PB pathway of grapevine infected by *L*. *theobromae* based on the FPKM value of transcripts.

The phenylpropanoid biosynthesis pathway, which is an important plant secondary metabolic pathway, plays significant roles in plant defense by enforcing or producing physical and chemical barriers against pathogen infection^43^. We identified 94 DEGs in the phenylpropanoid biosynthesis pathway in response to *L. theobromae* infection (Supplementary Table S11) and subsequently analyzed these DEGs using KEGG analysis (Fig. 5).

Phenylalanine ammonia-lyase (PAL), which is the first key enzyme in the phenylpropanoid pathway, is known to respond to pathogen infection. Out of a total of 18 transcripts encoding PAL, 16 were up-regulated upon *L*. *theobromae* infection. Furthermore, lignin is well characterized and plays an important role in defending against pathogens^44^. Here, in our data, a large number of enzymes associated with lignin biosynthesis were induced, including transcripts encoding cinnamyl-alcohol dehydrogenase (CAD), peroxidase (PO), 4-coumarate-CoA ligase (4CL), cinnamoyl-CoA reductase (CCR), caffeoyl-CoA O-methyltransferase, ferulate-5-hydroxylase (F5H) and trans-cinnamate 4-monooxygenase (CYP73A) (Fig. 5; Supplementary Table S8).

### Genes uniquely expressed in grapevine during *L. theobromae* infection

**Figure 6:**
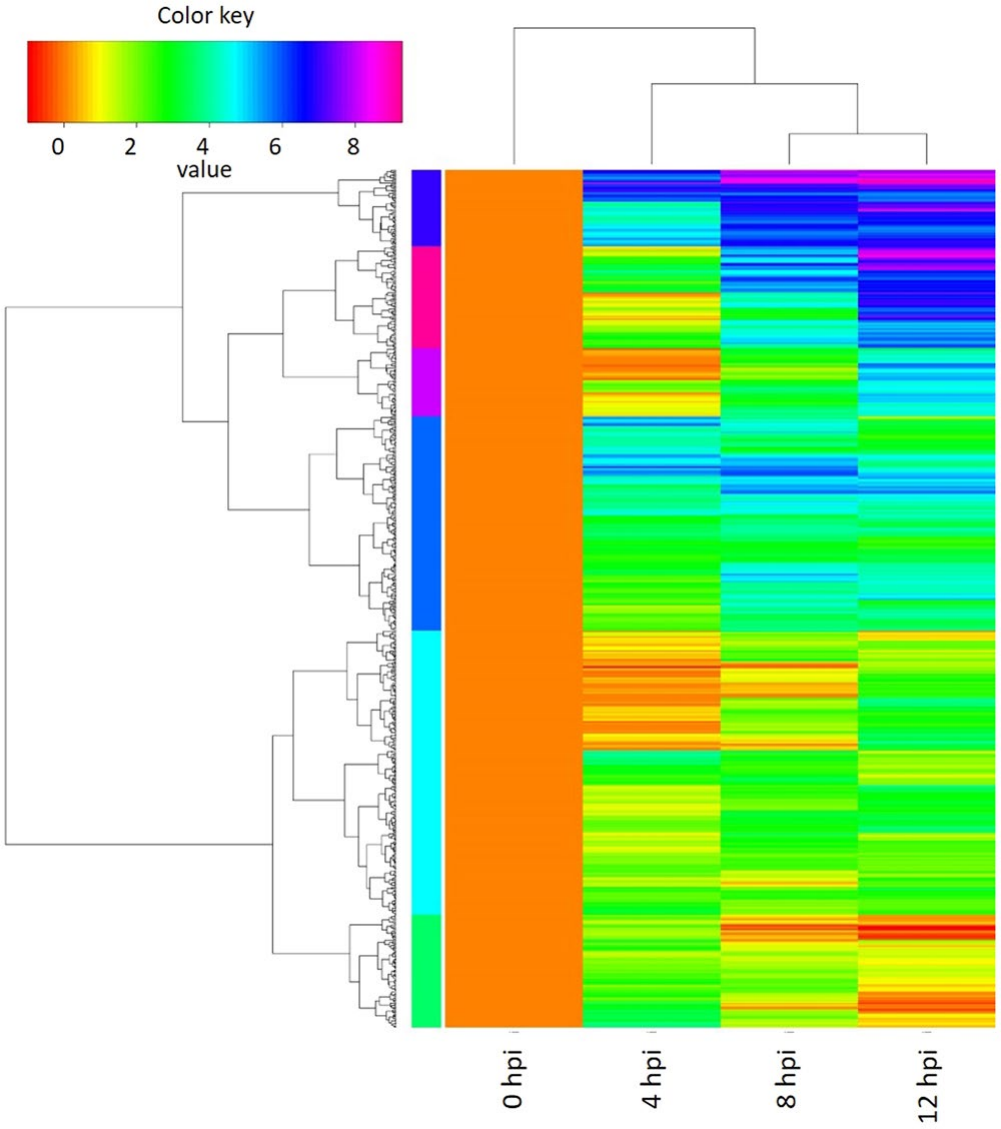
Hierarchical analysis of uniquely expressed genes (UEGs) in grapevine during *L*. *theobromae* infection. Genes with FPKM > 4 in any of the infected samples and FPKM < 1 in the 0 hpi samples were identified as UEGs.

The fragment per kilobase per million (FPKM) value of each transcript was estimated to evaluate the gene expression level. The genes of which FPKM was >4 in any of the infected samples and of which FPKM was <1 in the 0 hpi samples were identified as uniquely expressed genes (UEGs) upon *L. theobromae* infection. A total of 616 UEGs were identified, and we presume that these genes are those that are closely associated with systemic resistance (Fig. 6; Supplementary Table S12).

### Validation of the gene expression of DEGs by RT-qPCR

**Figure 7:**
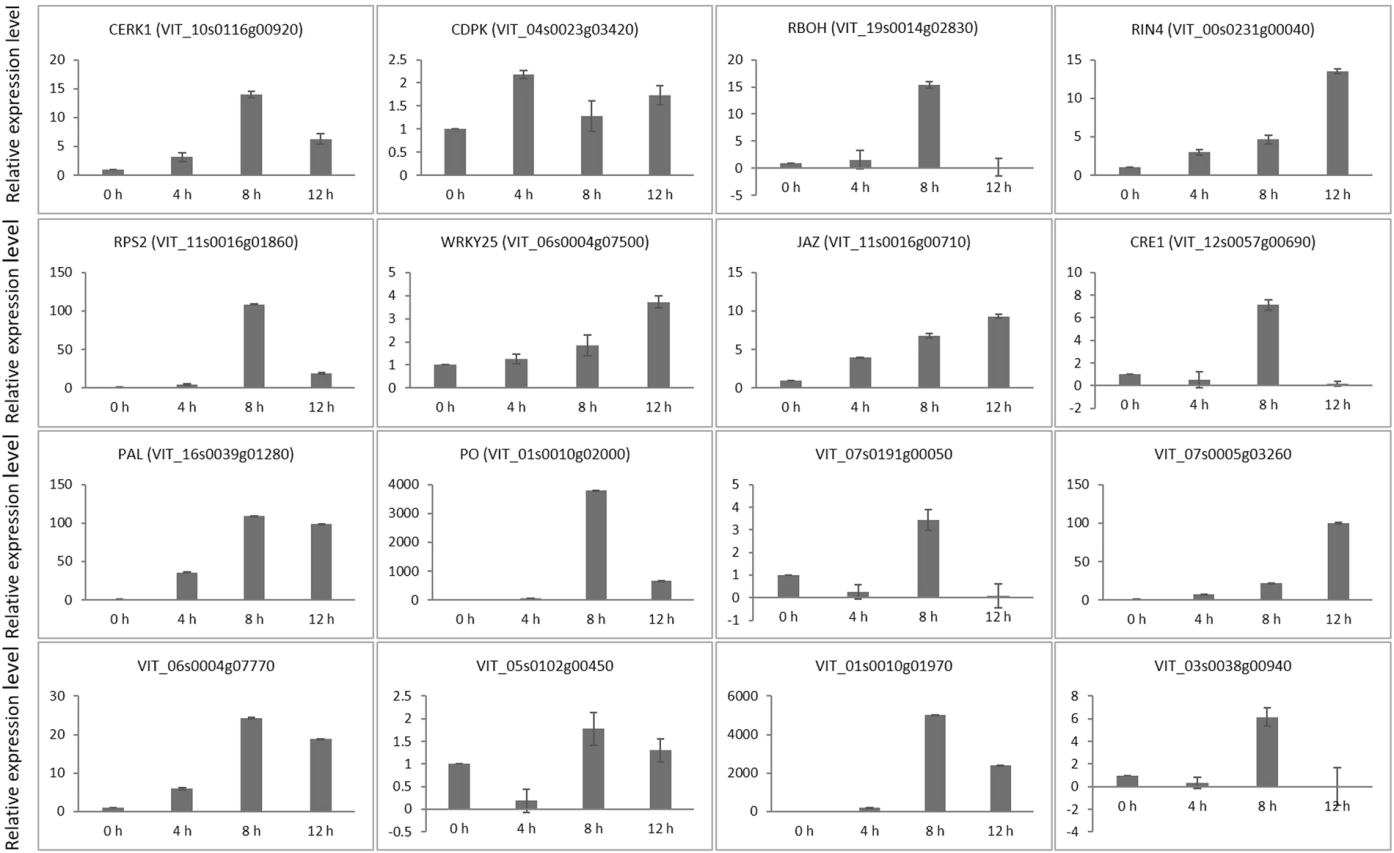
Validation of the gene expression of differentially expressed genes by reverse-transcriptase quantitative PCR. The gene expression levels of grapevine at different time points after *L*. *theobromae* infection are shown. The expression level of each gene was normalized to *Vvactin* and was set to 1 at 0 hours post-inoculation (hpi). Error bars represent the standard deviations (SDs) of two independent biological replicates and three technical replicates.

To validate the reliability of the RNA sequencing (RNA-seq) data, the relative expression level of 16 genes was confirmed using RT-qPCR with specific primers after 0, 4, 8, and 12 hpi (Fig. 7; Supplementary Table S13). Ten genes involved in the three key defense pathways (plant-pathogen interaction pathway, plant hormone signal transduction pathway, and phenylpropanoid biosynthesis pathway) and six randomly selected genes (VIT_07s0191g00050, VIT_07s0005g03260, VIT_06s0004g07770, VIT_05s0102g00450, VIT_03s0038g00940 and VIT_01s0010g01970) were used for validation analysis. The relative expression levels of these genes that were obtained from the RT-qPCR assays were compared with our RNA-seq data. The correlation coefficients for the different time points were calculated to assess the correlation between RNA-seq and RT-qPCR analyses. The correlation coefficient values were 0.948, 0.606 and 0.959 for 4, 8 and 12 hpi, respectively, and the correlation was significant at the 0.05 level (Supplementary Fig. S3). The RT-qPCR results showed that the gene expression level of the six randomly selected genes was induced at 4 hpi and reached their highest levels of expression at 8 hpi, whereas RIN4 and JAZ increased in expression over time (Fig. 7). These results further indicated that the plant innate immune response, phytohormone signaling transduction and secondary metabolism products might function together against infection by *L. theobromae* in grapevine.

## Discussion

Plants have evolved complicated signaling and defense pathways in response to pathogens over time. Identification and understanding of such processes will be a platform to develop genomic based breeding for economically important crops. Grapes have become one of the main fruits and beverage crops in China over the last few decades. Among several diseases associated with grapevines, the trunk disease caused by fungi belonging to the family Botryosphaeriaceae has a significant impact as it affects the perennial parts of the grapevine^1^. To date, the interaction mechanisms between grapevine and Botryosphaeria dieback are still poorly understood. Hence, the objectives of this study were to identify a set of genes responding to the early infection of *L*. *theobromae* and to understand signaling networks involved in grapevine. In this study, a total of 5181 DEGs were identified from the RNA-seq analysis performed, and KEGG analysis showed that pathways associated with primary metabolism, secondary metabolism, and defense response were enriched. Therefore, these results indicated that *L*. *theobromae* infection stimulated a wide range of responses in the host.

In plants, innate immunity, which consists of PTI and ETI, functions as the first line of defense responses^21,39^. FLS2 and CERK1, which are involved in the pathogen induced signal perception process, are two well-documented pattern recognition receptors (PRRs) located upstream of PTI. In the present study, CERK1 was up-regulated, whereas FLS2 was down-regulated after *L*. *theobromae* infection, indicating that CERK1 may mediate the plant immune pathway to restrain pathogen invasion; however, FLS2 may be recognized by an effector secreted by *L*. *theobromae*, leading to the suppression of *fls2* expression as *L*. *theobromae* releases many kinds of effectors to facilitate its infection process^18^. BAK1 and FLS2 were previously reported to form a flagellin-induced complex, which is essential for the downstream flg22-signaling responses in *Arabidopsis thaliana*^45^. In our study, BAK1 and five members of two independent pathogen-responsive MAPK cascades responded to *L*. *theobromae*, suggesting that there are other proteins besides FLS2 that form complexes with BAK1 and mediate downstream MAPK cascades. MEKK1, which is a negative regulator of defense responses, functions together with MKK1/2 and MPK4 to negatively regulate plant innate immune responses^46–48^. MPK3/6, which are downstream of MKK4/5, can act as positive regulators of defense responses^49,50^. In our study, the MKK1/2 and MPK4 encoding genes were down-regulated, whereas genes encoding MKK4/5 and MPK3/6 were up-regulated, followed by the increased expression of *WRKY25/33* and *WRKY22/29*, respectively, indicating that these two MAPK cascades participated in grapevine responses to *L*. *theobromae* infection. The up-regulated expression of *VvWRKY33* in grapevine responding to *L*. *theobromae* is consistent with the induction of these genes by powdery mildew^51^ and downy mildew^52^ in grapevine.

Calcium plays essential roles in regulating plant responses to fungi^53^. Ca^2+^ can activate CDPK and Rboh protein, which subsequently induces the production of ROS^54^. In grapevine, Ca^2+^ influx was previously proven to be one of the defense reactions when inoculated with *Botrytis cinerea*^55^. Our data showed that CNGCs, CDPK and Rboh genes were up-regulated (Fig. 4), indicating that Ca^2+^ plays crucial roles in signal transduction during the early stages of grapevine defense response.

To infect plants, some pathogens have evolved secreted effectors that suppress PTI^56^. In response to these effectors, plants employ ETI. Some NB-LRR domain-containing proteins in plants have been identified as receptor proteins that recognize the effector proteins^21,39^. One receptor protein, RIN4, which is a negative regulator, can be sensed by two R proteins, RPM1 and RPS2, in the form of phosphorylation and degradation^57^. In grapevine, the RIN4, RPM1 and RPS2 encoding genes were up-regulated in response to *L*. *theobromae* infection (Fig. 3). HSP90, SGT1 and RAR1 are important cytosolic chaperones of many R proteins and are involved in the plant innate immunity response by forming a molecular chaperone complex^58^. We found that HSP90 and SGT1 were up-regulated and RAR1 was down-regulated in grapevine responding to *L*. *theobromae*. In addition, protein kinase PBS1, which activates R protein RPS5 via cleavage by an effector^59^, and EDS1, which is one of the essential components of basal resistance to pathogens, were both down-regulated in response to infection. In general, our data reveal that innate immunity consists of a large network of early responses to infection with *L*. *theobromae*.

Secondary metabolites are widespread in plants and contribute to plant disease resistance by functioning as defenses or as signaling molecules^60^. For example, plant hormones are essential signaling molecules that participate in plant growth, development and defense response processes. In our study, a large number of DEGs were enriched in some plant hormone signal transduction pathways, including the auxin, cytokinin, JA, SA and ET signaling pathways, indicating that these pathways may be involved in the responses of grapevines to *L*. *theobromae* infection. SA triggered in grapevine was degraded by enzymes, salicylate hydroxylase and intradiol ring cleavage dioxygenase, produced by *L*. *theobromae*^61^, which could explain why this pathogen successfully infects grapevine despite the genes of the SA signaling pathway continually increased expressed. SA is mainly involved in defending against biotrophic pathogens, whereas JA and ET usually participate in restraining necrotrophic pathogens^62–64^. The antagonism of SA and JA has been reported in rice and Arabidopsis^65^. For example, SA-response genes were activated by overexpressing *OsNPR1*, while JA marker genes were suppressed simultaneously in rice^66^. However, our results showed that most DEGs of the SA and JA signal transduction pathways were up-regulated in grapevine after *L*. *theobromae* infection, suggesting that the interaction between grapevine and *L*. *theobromae* may have different mechanisms compared with those observed in rice and Arabidopsis. Furthermore, this finding proved that *L*. *theobromae* has some unique infection mechanisms compared with other plant pathogens. Auxin signaling is usually a defense response in the plant against necrotrophic fungi, whereas it does not show an obvious relation to the hemibiotrophic pathogens^67,68^. In our study, all DEGs detected in the auxin transduction pathway were up-regulated, suggesting that grapevine may regulate auxin signaling to defend against *L*. *theobromae*. We proposed that *L*. *theobromae* secretes effectors into grapevine cells to increase auxin level and finally facilitate its infection, a speculation based on how *Pseudomonas syringae* invades *A*. *thaliana*^67^. Cytokinins acting as plant growth regulators are also involved in plant defense response. Cytokinins have been shown to induce resistance in *A*. *thaliana* against *P*. *syringae* by modulating SA signaling^69^. Recent research has shown that cytokinin signaling is activated by the *P*. *syringae* type III effector HopQ1, which leads to the suppression of the *Arabidopsis* defense response^70^. Here, in our study, DEGs of the cytokinin signaling pathway were up-regulated, indicating that *L*. *theobromae* may interfere with grapevine innate immunity through the secreted effectors.

Phenylpropanoid compounds can preform or induce physical and chemical barriers against pathogen infection, as well as secrete signal molecules that can ultimately induce defense-related gene expression in plants^43^. In the phenylpropanoid biosynthesis pathway analyzed in this study, most of the genes encoding PAL and enzymes involved in lignin biosynthesis were up-regulated after *L*. *theobromae* infection (Fig. 5). These outcomes were consistent with a previous study in which the PAL and 4CL genes of grapevine cells showed increased expression at 6 hpi when treated with *Eutypa lata* filtrates^71^. Similar results were also reported in rice, in which all of the transcripts encoding PAL were up-regulated during the early infection stage of *Xanthomonas oryzae* pv. *oryzae*^72^. Moreover, our results were consistent with previous studies of Arabidopsis^73^ and therefore indicated that grapevine may employ the PB pathway to form lignin or other small molecules to limit *L*. *theobromae* infection. *Lasiodiplodia theobromae* eliminated phenylpropanoid precursors to evade the plant defense response^61^; thus, it does make sense that grapevine continuously up-regulated genes related to phenylpropanoid compound biosynthesis to prevent infection of this pathogen.

In conclusion, we identified DEGs in the green shoots of grapevine inoculated with *L*. *theobromae* and obtained detailed expression profiles of the grapevine response over time. We identified a total of 5181 DEGs, of which 2177 (4 hpi), 3453 (8 hpi) and 4661 (12 hpi) were differentially expressed relative to those of the control (0 hpi). DEGs enriched in plant–pathogen interactions, hormone signal transduction, and the phenylpropanoid biosynthesis pathways were further analyzed. In conclusion, our results suggest that innate immunity, phytohormone signaling and many phenylpropanoid compounds (which constitute a complex defense network in plants) are involved in the response to infection. This study shows - sketchy information of grapevine against *L*. *theobromae*, deepens our knowledge of the plant molecular response to opportunistic fungal pathogens and will contribute to a better understanding of the molecular interactions between grapevine and *L*. *theobromae*. Additionally, the findings of this study will accelerate the research process on the resistance of grapevine against *L*. *theobromae* and will provide a better understanding of grapevine defense responses against plant pathogens.

## Materials and Methods

### Pathogen infection manipulation

Pathogenicity tests were conducted on 1-year-old green shoots of the grape cultivar ‘Summer Black’. Shoots were pruned to ~30 cm long. To inoculate with *L. theobromae*, 4-mm diameter wounds were made using a cork borer on each shoot. This procedure was followed by placing a 4 mm agar plug containing the fungal isolate on the wound. The wound with an agar plug was then wrapped with Parafilm (Bemis, Oshkosh, WI, USA). Ten shoots were used as biological replicates. Sterile potato dextrose agar plugs were used as control (designated 0 hpi). Inoculated shoots were planted in sterilized soil in small nursery pots and incubated under moist conditions in an incubation chamber at 25 °C with an 8/16 light/dark cycle.

Samples were collected from the shoots at 0, 4, 8, and 12 hpi. Two pieces of 0.5 cm^2^ tissues were cut above and below the inoculation sites from each shoot separately using flame-sterilized blades and forceps. The collected tissues were flash-frozen with liquid nitrogen and then immediately stored at −80 °C until RNA was extracted. Two biological replicates were collected at each time point. To obtain enough RNA for subsequent analyses, ten green shoots were pooled as one replicate.

### Library construction and RNA-seq

An equal amount of collected tissues from each replicate per time point (0, 4, 8 and 12 hpi) was used for RNA extraction. Total RNA was extracted using TRIzol according to the manufacturer’s instructions (Invitrogen, Carlsbad, CA, USA). RNA integrity, purity, and concentrations were assessed using an Agilent 2100 Bioanalyzer (Agilent Technologies, Palo Alto, CA, USA) and a NanoDrop 2000 spectrophotometer (Thermo Fisher Scientific, Waltham, MA, USA). Purification of messenger RNA (mRNA), construction of complementary DNA (cDNA) libraries, end repair of cDNA, adapter ligation, and cDNA amplification were performed by following methodologies for preparing Illumina RNA-seq libraries of Novogene Bioinformatics Technology Co., Ltd. (Beijing, China). The final quantified libraries were sequenced on an Illumina HiSeq. 2500 (Illumina) to generate paired-end reads of 150 bp.

### Sequence alignment

Quality control of reads was assessed by running the FastQC program (v0.11.5), and Trimmomatic (v0.36)^74^ and low-quality sequences (phred ≤ 20, adapter and poly-N contamination) were filtered out. All subsequent analyses used only the high-quality reads. The reference genome and gene model annotation files were downloaded from the grape genome website (ftp://ftp.ensemblgenomes.org/pub/release-23/plants/fasta/vitis_vinifera/dna/). After building the index of the reference genome using Bowtie v2.2.3 (https://sourceforge.net/projects/bowtie-bio/files/bowtie2/2.3.3/, Broad Institute, Cambridge, MA, USA), paired-end reads were aligned to the reference genome using TopHat v2.0.12 (http://ccb.jhu.edu/software/tophat/manual.shtml, Broad Institute), and HTSeq v0.6.1 (https://files.pythonhosted.org/packages/3c/6e/f8dc3500933e036993645c3f854c4351c9028b180c6dcececde944022992/HTSeq-0.6.1p1.tar.gz, EMBL, Heidelberg, Germany) was used to count the read numbers mapped to each gene. The Cufflinks v2.1.1 (http://cole-trapnell-lab.github.io/cufflinks/) Reference Annotation Based Transcript (RABT) assembly method was used to construct and identify both known and novel transcripts from TopHat alignment results^75,76^. The reads per kilobase of transcript per million mapped reads (RPKM) of each gene was calculated based on the length of the gene and read count mapped to it. We carried out read alignment and expression quantification separately for each sample. Only genes with fragments per kilobase of transcript per million mapped reads (FPKM) that had a value >4 and that exhibited low variation across two biological replicates (coefficient of variation <30%) were considered reliable and were used in subsequent analyses^77^.

### Identification and functional enrichment analyses of DEGs

The identification of DEGs among the six treatments was performed using the DESeq R package v1.18.0, which provides statistical routines for determining differential expression in digital gene expression data using a model based on the negative binomial distribution. The resulting p-values were adjusted using Benjamini and Hochberg’s approach to control the false discovery rate (FDR)^78^. Genes with an adjusted p-value < 0.05 found by DESeq. 2 were assigned as differentially expressed.

### GO and KEGG pathway analysis

Gene Ontology (GO) enrichment analysis of differentially expressed genes was implemented by the clusterProfiler R package, in which gene length bias was corrected. GO terms with corrected p values less than 0.05 were considered as significantly enriched DEGs. To understand the molecular functions of DEGs, the clusterProfiler R package used to test the statistical enrichment of DEGs in KEGG pathways(http://www.genome.jp/kegg/).

### Cluster analysis of gene expression differences

Cluster analysis is used to find genes with similar expression patterns under various experimental conditions. By clustering genes with similar expression patterns, it is possible to discern unknown functions of previously characterized genes or functions of unknown genes. In hierarchical clustering, areas of different colors denote different groups (clusters) of genes, and genes within each cluster may have similar functions or take part in the same biological process. In addition to the FPKM cluster, the H-cluster with complete linkage method was also used to cluster the log2 fold change. Genes within the same cluster exhibit the same trends in expression levels under different conditions.

### Real-time qPCR analysis

To validate the RNA-seq results, we performed reverse-transcriptase quantitative PCR (RT-qPCR) using the same biological replicates. We treated 1 µg of RNA with DNase I (Thermo Fisher Scientific) to remove DNA before using SuperScript IV Reverse Transcriptase (Thermo Fisher Scientific) to perform a cDNA synthesis reaction according to the manufacturer’s protocols. A total of 16 genes were selected for qPCR validation of the RNA-seq results. The grapevine actin gene (Vvactin, accession D28123) was used as an endogenous control to normalize the gene expression data. Primers were designed using the Primer3 web tool (v0.4.0, http://bioinfo.ut.ee/primer3-0.4.0/). All primers are listed in Supplementary Table S14. RT-qPCR was performed on an ABI 7500 real-time PCR system (Applied Biosystems, Carlsbad, CA, USA) using SYBR^®^ Premix Ex Taq™ (Tli RNaseH Plus) (Takara Bio Inc., Kusatsu, Shiga, Japan) according to the manufacturer’s instructions. Two biological replicates were used for RT-qPCR analysis for each treatment. At the same time, three technical replicates were used for each biological replicate. The ΔΔCT method was used for relative quantification of gene expression^79^. The standard deviations (SDs) and correlation coefficients were analyzed by Microsoft Excel 2016 (Microsoft Corporation, Redmond, WA, USA) and IBM SPSS Statistics 21.0 software (IBM China Company Ltd., Beijing, China), respectively.

## Supplementary information

Figure S1-S3

Table S1-S13

## Data Availability

Supplementary materials are uploaded with the manuscript, and they are available from the corresponding author upon reasonable request.
