# Supplementary material for: Transcriptional response of grapevine to infection with the fungal pathogen Lasiodiplodia theobromae
Source: Sci Rep. 2019 Mar 29;9:5387. doi: 10.1038/s41598-019-41796-9 (PMC6441073; doi:10.1038/s41598-019-41796-9)
Supplement: Supplementary file 1 — Figure S1-S3 [file 41598_2019_41796_MOESM1_ESM.docx]

Transcriptional response of grapevine to infection with the fungal pathogen *Lasiodiplodia theobromae*

Wei Zhang^1,2,3^, Jiye Yan^2,3^, Xinghong Li^2,3^, Qikai Xing^2,3^, K. W. Thilini Chethana^2,3^ & Wensheng Zhao ^1,^ *

**Supplementary figures**


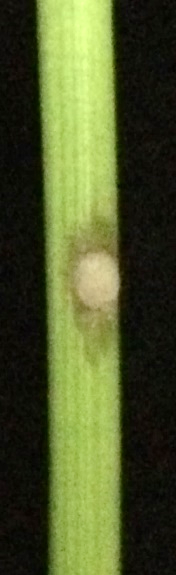

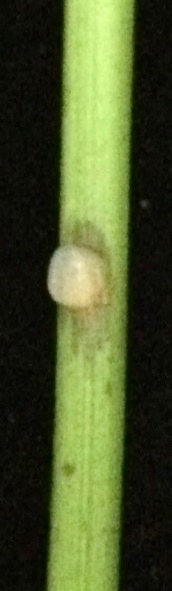


12 hpi


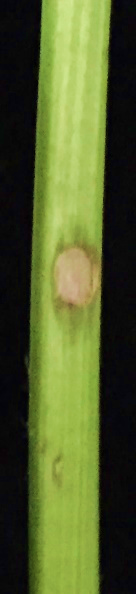

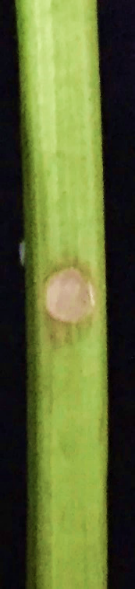


4 hpi


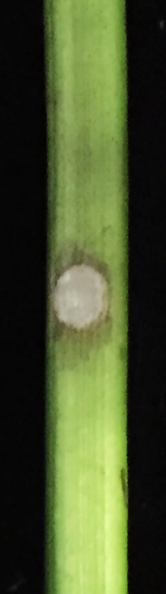

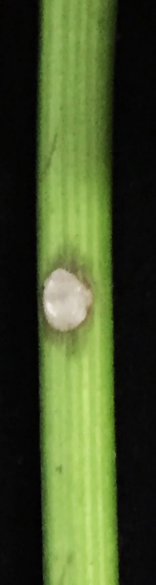


8 hpi


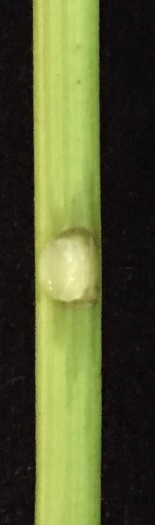

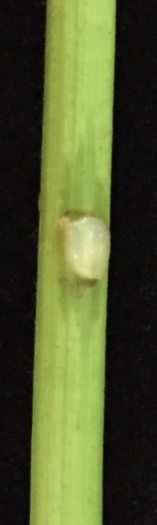


0 hpi

**Figure S1.** Grapevine green shoots infected with *L. theobromae* after different inoculation time point


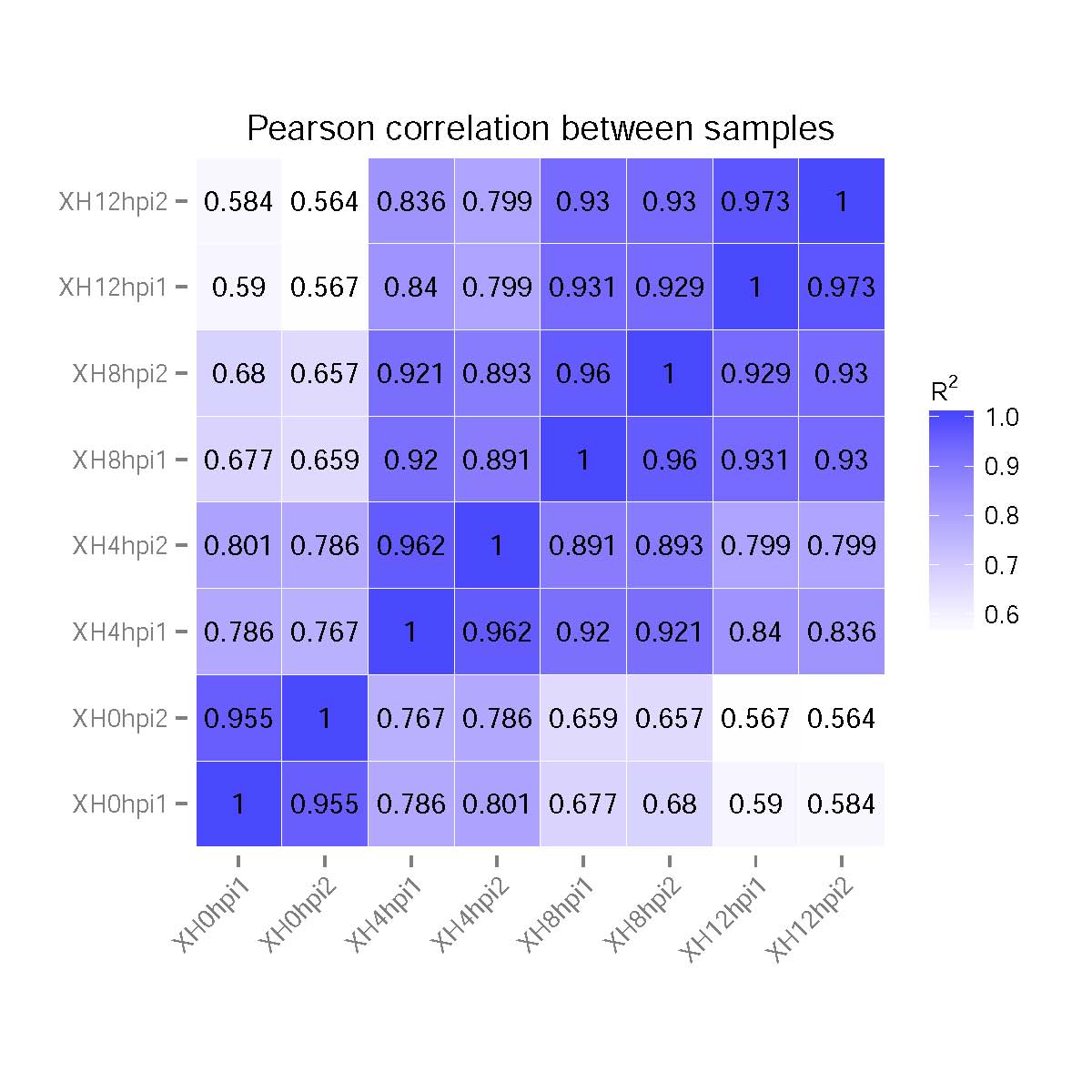


**Figure S2.** Pearson correlation between different grapevine green shoots samples infected with *L. theobromae* at different time points


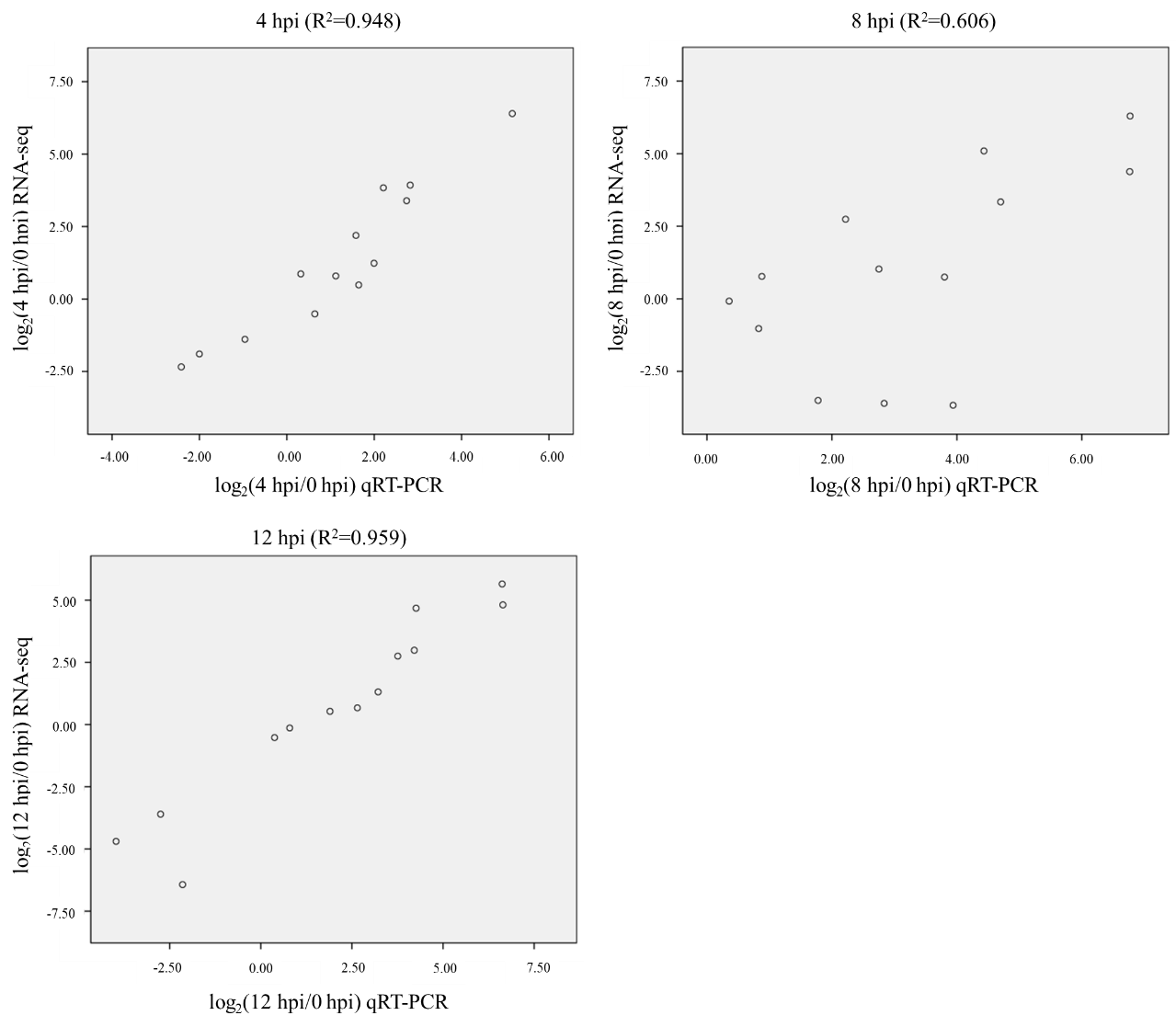


**Figure S3.** Correlation between RNA-seq and qRT-PCR data at different time points
